# HDR Scene Reconstruction from Resolution-Mismatched Event Streams and Single-Exposure Images

**DOI:** 10.3390/s26144621

**Published:** 2026-07-21

**Authors:** Zehao Chen, Binbin Zhou, Zengwei Zheng

**Affiliations:** School of Computer and Computing Science, Hangzhou City University, Hangzhou 310015, China

**Keywords:** event camera, high dynamic range imaging, 3D Gaussian Splatting, neural radiance field, multi-view reconstruction

## Abstract

High dynamic range (HDR) radiance-field reconstruction from single-exposure low dynamic range (LDR) images is limited by the information loss in saturated regions, while event cameras provide complementary measurements whose dynamic range far exceeds that of conventional sensors. In practical hybrid sensor systems, however, the RGB camera usually has a higher spatial resolution than the event sensor, which makes existing event-aided HDR reconstruction methods difficult to apply directly. A straightforward solution is to super-resolve the event stream before reconstruction, but this 2D preprocessing introduces a global color cast and view-inconsistent high-frequency artifacts once the super-resolved events supervise a 3D radiance field. We propose a framework for HDR radiance-field reconstruction from resolution-mismatched event–image inputs. The framework incorporates a pretrained 2D event super-resolution prior and corrects its transfer to 3D reconstruction through a color correction module, which anchors the rendered radiance to the chrominance of the LDR images, and a dual-resolution event-stream constraint, which supervises the synthesized events at both the super-resolved and the native resolution. Experiments on the EvHDR-NeRF benchmark show that the proposed method achieves higher six-scene mean HDR fidelity than the strongest baseline while reducing the global color cast and alleviating multi-view artifacts.

## 1. Introduction

High dynamic range (HDR) reconstruction is a fundamental problem in computer vision and graphics, with broad applications in film production, virtual and augmented reality, autonomous driving, and computational photography. Traditionally studied in the image and video domains, HDR reconstruction has recently shifted to the scene domain through neural representations such as Neural Radiance Fields (NeRF) [1] and 3D Gaussian Splatting (3DGS) [2]. Compared with image- and video-domain HDR, scene-level HDR reconstruction synthesizes HDR images at arbitrary novel viewpoints, supporting downstream tasks such as free-viewpoint relighting [3], AR/VR insertion [4], and physics-based simulation [5].

Existing HDR scene reconstruction methods are either multi-exposure image-based or event-based, according to their input modality. Multi-exposure image-based methods [6,7,8,9] require multiple exposure levels across viewpoints, which limits their applicability in practical capture scenarios. Event-based methods [10,11,12] exploit the high dynamic range and high temporal resolution of event cameras [13] to recover an HDR scene from a single LDR exposure per viewpoint together with an event stream. They place only mild requirements on the LDR input and thus substantially broaden the range of applicable capture scenarios.

**Figure 1:**
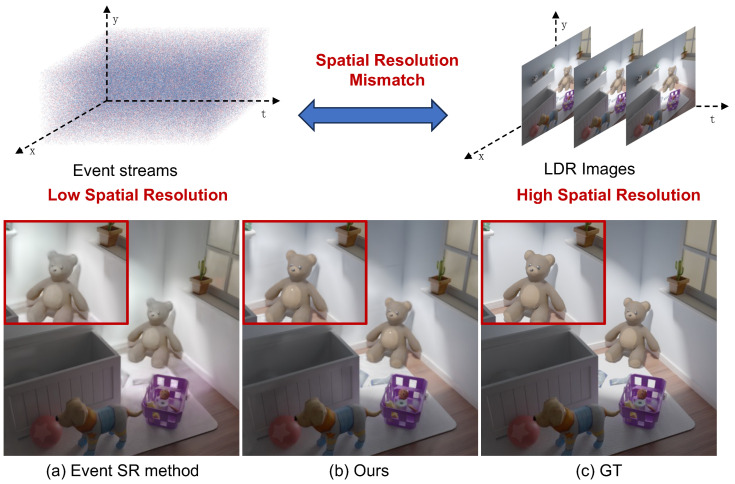
The resolution mismatch between a low-resolution event stream and high-resolution single-exposure LDR images (top). (**a**) A baseline that super-resolves the events with a 2D network and feeds them to an event-aided HDR pipeline suffers a global color cast and washed-out, multi-view-inconsistent details. (**b**) Our framework restores faithful color and sharp detail through the color correction module (Section 4.2.2) and the dual-resolution event-stream constraint (Section 4.2.3), closely matching (**c**) the ground truth.

In current practice, event signals and image signals are commonly acquired through hybrid camera rigs that combine an RGB camera with an event camera [14,15]. Due to hardware constraints of the two sensors, the event resolution is typically lower than the image resolution. This resolution mismatch challenges high-resolution HDR scene reconstruction. The low-resolution event stream supplies only coarse gradient information, so high-frequency content of the underlying HDR scene cannot be recovered and the reconstructed field loses fine-scale structure (Figure 1).

A natural remedy is to super-resolve the low-resolution event stream before applying an event-aided HDR reconstruction pipeline. However, existing event super-resolution methods [16,17,18] are formulated as per-frame image-plane operations on event polarity alone. They neither account for the geometric relationship across viewpoints nor model how the super-resolved events should distribute over the RGB channels of the underlying scene. As a consequence, 2D event super-resolution is not constrained by the color distribution or the multi-view geometry of the target radiance field, and when used as 3D supervision, the super-resolved events induce a global color bias and view-inconsistent high-frequency artifacts (Section 5.3). Transferring event super-resolution priors into 3D HDR reconstruction therefore requires compensating, on the 3D side, for the color and multi-view consistency that the 2D super-resolution stage does not provide.

In this paper, we propose a framework for HDR scene reconstruction from resolution-mismatched event–image inputs. The framework takes a low-resolution event stream paired with high-resolution single-exposure LDR images and recovers an HDR radiance field at the image resolution, without requiring the two sensors to share the same resolution. To bridge the resolution mismatch, we adopt a 2D event super-resolution network as a learned prior that lifts the low-resolution event stream to the image resolution and supervises the 3D HDR scene reconstruction pipeline. Because this prior is trained with 2D image-plane objectives rather than 3D reconstruction constraints, its transfer into 3D HDR reconstruction exposes two failure modes. The per-channel intensity distribution of the super-resolved events drifts and propagates into a global color cast in the reconstructed radiance field, and the high-frequency content they add is inconsistent across viewpoints and appears as artifacts in novel views. To address these failure modes, we introduce two complementary modules. The first is a color correction module that anchors the rendered radiance to the color information of the LDR images through a chrominance consistency constraint and a response-curve regularizer, removing the global color cast that the super-resolved events introduce. The second is a dual-resolution event-stream constraint that supervises the synthesized events at the super-resolved resolution and the native sensor resolution simultaneously, encouraging the radiance field to retain the high-frequency content that is consistent across the two resolutions and alleviating the multi-view artifacts left by the 2D super-resolution objective. Extensive experiments on Synthetic HDR scenes show that the proposed framework achieves higher HDR fidelity than strong event-aided HDR baselines under the resolution-mismatched setting while also improving color neutrality and perceptual quality.

The main contributions of this work are as follows:

- To our knowledge, we propose the first framework for HDR scene reconstruction from resolution-mismatched event–image inputs, which transfers the prior learned by a 2D event super-resolution network into the 3D HDR scene reconstruction pipeline.
- We introduce a color correction module that anchors the rendered radiance to the chrominance of the LDR observations through a chrominance consistency constraint and a response-curve regularizer, reducing the global color cast introduced by the super-resolved events.
- We propose a dual-resolution event-stream constraint that supervises the synthesized events at both the super-resolved and the native event resolution, alleviating the view-inconsistent high-frequency artifacts introduced by 2D event super-resolution.

## 2. Related Work

### 2.1. HDR Radiance Field Reconstruction

Recovering the high dynamic range of a scene from multi-view observations originates in classic multi-exposure HDR imaging, which calibrates the camera response and fuses bracketed exposures into a single HDR image [19,20,21]. With the introduction of NeRF [1] and 3DGS [2], existing works reformulate the problem as fitting an HDR radiance field to multi-view LDR observations, and can be grouped by the exposure setting of the inputs. Multi-exposure methods follow the classic recipe and invert the camera response from an exposure stack. HDR-NeRF [6] recovers the HDR radiance through an explicit response model, HDR-Plenoxels [22] jointly calibrates the camera response and the exposure ratios, and RawNeRF [7] avoids response inversion by training directly on raw sensor data. HDR-HexPlane [8] extends the formulation to dynamic scenes. HDR-GS [9], HDRGS [23], and HDRSplat [24] transfer the multi-exposure recipe to 3D Gaussian Splatting; Cinematic Gaussians [25] add depth-of-field and exposure control; and Casual3DHDR [26] relaxes the capture assumption to casually recorded video. Single-exposure methods drop the exposure stack. GaussHDR [27] fits an HDR field to one exposure per viewpoint through a learned tone mapping with uncertainty weighting, SeHDR [28] synthesizes a virtual exposure bracket from the single-exposure input, and related 3DGS variants [29] explore similar setups.

Existing works in both groups assume that all image observations share a single spatial resolution, so resolution alignment never arises. In contrast, our setting assumes only single-exposure LDR images at each viewpoint and relies on a lower-resolution event stream to recover the saturated HDR information, which requires the resolution mismatch to be handled inside the 3D reconstruction rather than as an isolated preprocessing step.

### 2.2. Event-Aided 3D Reconstruction

Event cameras [13] asynchronously record per-pixel logarithmic brightness changes with a dynamic range exceeding 120 dB and microsecond-level latency, and are therefore well suited to recovering radiance in the regions where conventional sensors saturate. Existing works integrate events into radiance field reconstruction along two lines. The first line reconstructs the scene from events alone. EventNeRF [30] and EvNeRF [31] learn a radiance field whose log-radiance differences between temporally adjacent views match the event stream, Klenk et al. [32] extend the formulation with motion-compensated event batching, ENeMF [33] models the event motion explicitly, Deblur e-NeRF [34] accounts for the pixel bandwidth of the sensor, and AE-NeRF [35] targets non-ideal capture conditions. Event-3DGS [36] and EventSplat [37] fit 3D Gaussian fields directly to the event stream. The second line combines events with RGB images to exploit the dynamic range of the former and the color fidelity of the latter. EvHDR-NeRF [10] and EvHDR-GS [11] reconstruct HDR radiance fields from single-exposure RGB images and co-temporal event streams. E2NeRF [38], Ev-DeblurNeRF [39], EBAD-NeRF [40], EaDeblur-GS [41], and EBAD-Gaussian [42] use events to recover sharp scenes from motion-blurred inputs. Further event–RGB fusion strategies [43,44,45,46] and dynamic-scene extensions [12,47,48] extend this family.

Existing works implicitly assume that the event stream and the RGB images share the same spatial resolution, either by synthesizing the dataset at matching resolutions or by relying on a co-located sensor like DAVIS [49] that limits the RGB channel to the native event resolution. Practical hybrid camera systems [14,15], however, pair a high-resolution RGB camera with a separate, lower-resolution event sensor and thus violate this assumption. Our work reconstructs an event-aided HDR radiance field under this resolution-mismatched setting.

### 2.3. Event-Based Super-Resolution

A natural way to handle the event–image resolution mismatch is to super-resolve the low-resolution event stream before reconstruction. Existing event super-resolution works treat this task in the 2D image domain and follow two threads. The first thread super-resolves the event stream alone. Early formulations [50] treat the event field as a single-channel image and apply convolutional upsamplers, EventZoom [16] jointly denoises and super-resolves the stream, EventSR [17] adopts adversarial training on real event data, Mostafavi et al. [51] model event-specific noise statistics, and Weng et al. [52] aggregate temporal context through a recurrent model. BMCNet [18] mines bidirectional motion cues across consecutive event frames and represents the current state of the art; we adopt it as the super-resolution prior in our framework. Li et al. [53] study generalization across sensors with different contrast thresholds, and spiking formulations [54,55] pursue lightweight inference. The second thread conditions the upsampling on auxiliary intensity frames. Guo et al. [56] use events to guide image super-resolution under low light, Zhang et al. [57] fuse events with blurry RGB inputs, and EvTexture [58] uses events to enhance the texture of video super-resolution.

Existing works in this family design and evaluate the super-resolution network against 2D image-domain fidelity targets, without regard for any downstream 3D reconstruction task. They provide useful 2D priors, but they enforce neither the per-pixel channel assignment of the color filter array nor the multi-view geometric consistency required by 3D HDR reconstruction, and the super-resolved events consequently induce a global color cast and view-inconsistent high-frequency artifacts in the reconstructed field. Our work introduces the super-resolution prior into 3D HDR reconstruction together with a color correction module and a dual-resolution event-stream constraint that address these two failure modes.

## 3. Preliminaries

### 3.1. HDR 3DGS

We build on 3D Gaussian Splatting (3DGS) [2], which represents a scene as a set of anisotropic Gaussian primitives

(1) $$G={\left\{\left(\mu_{i},\;\Sigma_{i},\rho_{i},\;c_{i}\right)\right\}}_{i=1}^{N},$$

where $\mu_{i}\;\in \;R^{3}$ is the centre, $\Sigma_{i}\;\in \;R^{3\times 3}$ the covariance, $\rho_{i}\;\in \;\left[0,1\right]$ the opacity, and $c_{i}$ the view-dependent radiance descriptor of the *i*-th primitive. The color at pixel x is rendered by front-to-back alpha compositing,

(2) $$c\left(x\right)=\sum_{i=1}^{N}c_{i}\;\rho_{i}\;G_{i}^{2D}\left(x\right)\prod_{j=1}^{i-1}\left(1-\rho_{j}G_{j}^{2D}\left(x\right)\right),$$

where $G_{i}^{2D}$ is the screen-space projection of the *i*-th primitive. HDR Gaussian Splatting [9] treats the descriptor $c_{i}$ as a scene-referred HDR radiance, so that Equation (2) renders an HDR radiance image that a camera response function maps to the LDR domain for supervision. Our framework follows this HDR rendering paradigm and uses GaussHDR [27], which realizes the response mapping with a learned tone-mapping head; we write ${\hat{I}}_{v}^{\mathrm{HDR}}$ for the HDR radiance rendered at viewpoint *v* through Equation (2).

### 3.2. Event and Image Formation Model

We adopt the event and image formation model of [10]. The event signal is generated as

(3) $$e\left(u,t\right)=\lfloor \left(\log L\left(u,t\right)-\log L\left(u,t-\Delta t\right)\right)/C_{\mathrm{thr}}\rfloor ,$$

where L(u,t) is the instantaneous radiance at pixel u=(u,v) and time *t*, Δt is the interval since the last event at the same pixel, and $C_{\mathrm{thr}}$ is the contrast threshold. The brackets ⌊·⌋ denote the floor function, which models the quantization of the log-radiance change by the contrast threshold $C_{\mathrm{thr}}$. The per-channel HDR frame is the temporal integral of the radiance over the exposure of duration *T*,

(4) $$I_{\mathrm{HDR},c}\left(u\right)=\int_{t-T}^{t}L_{c}\left(u,\tau \right)\;d\tau ,\;c\in \left\{R,G,B\right\},$$

and is mapped to its displayed LDR counterpart by a learnable per-channel camera response function (CRF) $f_{c}\left(\cdot \right)$ in the log-radiance domain,

(5) $$I_{\mathrm{LDR},c}\left(u\right)=f_{c}\;\left(\log I_{\mathrm{HDR},c}\left(u\right)\right)=f_{c}\;\left(\log L_{c}\left(u\right)+\log T\right),$$

where the three channel responses $f_{R},f_{G},f_{B}$ are parameterized independently, a property our color-correction module relies on. We write $I_{\mathrm{LDR}}=f_{c}\left(I_{\mathrm{HDR}}\right)$ for the channel-wise mapping.

## 4. Proposed Method

We aim to reconstruct a high-resolution HDR radiance field from a resolution-mismatched hybrid capture. The input consists of a low-resolution event stream $E^{L}\in R^{H_{L}\times W_{L}}$ at the native sensor resolution and a set of high-resolution single-exposure LDR images. The output is a 3D Gaussian Splatting representation G from which novel-view HDR and LDR images can be rendered at the image resolution.

Section 4.1 models the event–image relationship under resolution mismatch. Section 4.2 presents the framework built on this relationship, together with the two corrective modules that make the 2D super-resolution prior compatible with the 3D radiance field. Section 4.3 describes the optimization.

### 4.1. Modeling the Event–Image Relationship

Existing works [10] model the event–image relationship through the camera response function and the exposure time, but do not consider the case where the events and the images have different resolutions. In practice, however, the two signals are captured by different sensing devices whose resolutions are generally not the same, which limits the applicability of this formulation. We extend the event–image formation model by explicitly incorporating the resolution mismatch between the two sensors:

(6) $$\begin{array}{cc} E^{L}\left(u\right)\; & =\lfloor \left(\log D(L(u,t))-\log D(L(u,t-\Delta t))\right)/C_{\mathrm{thr}}\rfloor , \end{array}$$

(7) $$\begin{array}{cc} I_{\mathrm{LDR}}\left(u\right)\; & =f_{c}\;\left(\bar{L}\left(u\right)\cdot T\right), \end{array}$$

where $E^{L}$ is the native event stream at resolution $H_{L}\times W_{L}$; $D:R^{H\times W}\;\to \;R^{H_{L}\times W_{L}}$ is the spatial downsampling operator that maps the image-resolution radiance to the event resolution; L(u,t) is the HDR radiance at pixel u and time *t*; $C_{\mathrm{thr}}$ is the event contrast threshold; Δt is the time interval between the two adjacent instants of the event stream; *T* is the exposure time; and $I_{\mathrm{LDR}}$ is the LDR intensity under the per-channel camera response $f_{c}$. The time-averaged radiance is $\bar{L}\left(u\right)=\frac{1}{T}\int_{t-T}^{t}L\left(u,\tau \right)\;d\tau$; for the benchmark’s static scenes the radiance is constant over the exposure interval, so this reduces to L·T. The two observations are thus tied to the same underlying radiance *L*, with the resolution mismatch made explicit by D.

Recovering image-resolution event information can therefore be viewed as approximating the inverse of the downsampling operator D. We denote this super-resolution operator by $S:R^{H_{L}\times W_{L}}\;\to \;R^{H\times W}$. Existing event super-resolution methods [16,18,52] learn S in the 2D image plane under per-frame fidelity objectives, super-resolving the event stream independently at each time step, and therefore cannot guarantee that the super-resolved event stream preserves the color distribution and the multi-view consistency of the original stream. The key challenge is thus to introduce such a super-resolution prior without violating the color and multi-view consistency required by the 3D radiance field. We propose two modules that handle the color and the multi-view problems respectively, enabling existing 2D priors to be applied to 3D HDR radiance field reconstruction.

### 4.2. Framework

In this section, we first introduce the overall framework. The framework takes the low-resolution event stream together with the high-resolution single-exposure LDR images and recovers an HDR radiance field at the image resolution, where the super-resolution operator S introduced in Section 4.1 is provided by a pretrained 2D event super-resolution prior. To make the 2D prior compatible with 3D HDR reconstruction, we then introduce two corrective modules, one enforcing color consistency and one enforcing dual-resolution event consistency.

#### 4.2.1. Overview

**Figure 2:**
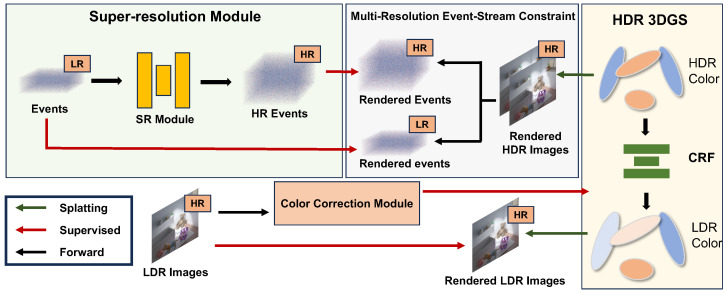
Overview of the proposed framework. A pretrained 2D event super-resolution prior S super-resolves the native low-resolution event stream $E^{L}$ to the image resolution ($E^{H}$). The scene is represented by HDR Gaussian primitives and rendered at each sampled viewpoint into an HDR image ${\hat{I}}_{v}^{\mathrm{HDR}}$ and its tone-mapped LDR prediction ${\hat{I}}_{v}^{\mathrm{LDR}}$. The color correction module anchors the chrominance of the rendered radiance to the LDR observations, and the dual-resolution event-stream constraint supervises the synthesized events at the super-resolved resolution and the native resolution simultaneously.

The framework is illustrated in Figure 2. The scene is represented by HDR Gaussian primitives [27] G. The HDR image at a novel viewpoint *v* can be obtained through differentiable splatting,

(8) $${\hat{I}}_{v}^{\mathrm{HDR}}=R\left(G,v\right),$$

where R denotes the alpha-compositing splatting renderer of Equation (2). Following the event generation model of Equation (3), the accumulated events between two adjacent time instants $t_{1}$ and $t_{2}$ are synthesized from the corresponding rendered HDR images,

(9) $${\hat{E}}_{t_{1}\to t_{2}}^{H}\left(u\right)=\log{\hat{I}}_{t_{2}}^{\mathrm{HDR}}\left(u\right)-\log{\hat{I}}_{t_{1}}^{\mathrm{HDR}}\left(u\right).$$

The real events are used to supervise this radiance field. Over the same interval, the accumulated real events are obtained from the event stream as

(10) $$E_{t_{1}\to t_{2}}\left(u\right)=C_{\mathrm{thr}}\sum_{t\in (t_{1},\;t_{2}]}e\left(u,t\right).$$

The synthesized events, however, lie at the image resolution H×W, whereas the real events $E^{L}$ lie at the sensor resolution $H_{L}\times W_{L}$. The two are therefore related through the super-resolution operator S, which lifts the native stream to the image resolution, $E^{H}=S\left(E^{L}\right)$. We represent S with a pretrained 2D event super-resolution network applied once per scene at the input stage, thereby introducing the super-resolution prior into the reconstruction.

Because the pretrained model [16,18,52] is optimized in the 2D image plane, it does not explicitly enforce the constraints required by 3D radiance-field optimization. The super-resolved stream therefore neither preserves the color distribution of the scene nor stays consistent across viewpoints, which leads to a global color cast and view-inconsistent high-frequency artifacts in the reconstructed radiance field. The color correction module anchors the rendered radiance to the chrominance of the LDR images, and the dual-resolution event-stream constraint supervises the rendering against the native stream $E^{L}$ in parallel with $E^{H}$, filtering out the view-inconsistent component. Both corrections are implemented as loss terms and require no architectural change to the Gaussian representation.

#### 4.2.2. Color Correction Module

The event stream records the color information of the scene through an RGBG Bayer pattern [59]. Each pixel responds to a single color channel determined by the color filter array, so the channel identity of an event is tied to its pixel position. This assumption applies specifically to color event sensors equipped with a color filter array, such as the Color-DAVIS346 [59], and is not a property of event cameras in general, many of which are monochrome. Because the proposed color correction formulation relies on the channel-dependent event information provided by the RGBG Bayer pattern, the current method is not directly applicable to monochrome event cameras; extending it to such sensors would require a different event–image formation model and corresponding modifications to the color correction mechanism. Existing event super-resolution works [16,18,52] model the spatio-temporal relations among the events but ignore this structural relationship between different pixels. The super-resolved event stream therefore no longer respects the per-pixel channel assignment, and under its supervision the per-channel intensities of the rendered radiance drift away from the actual scene chrominance. This channel-wise drift propagates through optimization and appears as a global color cast in the reconstructed HDR radiance field.

We mitigate this drift by anchoring the rendered radiance to the chrominance information preserved in the LDR images through two complementary constraints, acting on the displayed chrominance and on the camera response curves. Let ${\hat{I}}_{v}^{\mathrm{LDR}}$ denote the LDR prediction obtained by applying the per-channel responses $f_{c}$ to the HDR image rendered at image-pose *v*, and $I_{v}^{\mathrm{LDR}}$ the LDR observation at the same pose.

The first constraint matches the chrominance of the two images over non-saturated pixels:

(11) $$L_{\mathrm{chr}}=\frac{1}{|\Omega_{v}^{\mathrm{ns}}|}\sum_{u\in \Omega_{v}^{\mathrm{ns}}}∥\phi \left({\hat{I}}_{v}^{\mathrm{LDR}}\left(u\right)\right)-\phi \left(I_{v}^{\mathrm{LDR}}\left(u\right)\right){∥}_{1},$$

where $\phi :R^{3}\;\to \;R^{2}$ is a fixed color transform that discards the luminance component of an RGB color and retains its two chrominance components, and $\Omega_{v}^{\mathrm{ns}}$ is the set of pixels at which $I_{v}^{\mathrm{LDR}}$ is not saturated. Saturated pixels carry no reliable chrominance and are excluded. The second constraint addresses the per-channel responses $f_{R},f_{G},f_{B}$ of Equation (5), whose independent parameterization allows the three curves to drift apart and re-introduce a channel offset downstream of the anchored radiance. We regularize the three curves toward a shared response over the log-radiance domain:

(12) $$L_{\mathrm{crf}}=\frac{1}{3}\sum_{(c_{1},c_{2})\in P}\frac{1}{M}\sum_{m=1}^{M}{\left(f_{c_{1}}\left(x_{m}\right)-f_{c_{2}}\left(x_{m}\right)\right)}^{2},$$

where ${\left\{x_{m}\right\}}_{m=1}^{M}$ are *M* points sampled uniformly over the log-radiance range and P={(R,G),(G,B),(R,B)} enumerates the channel pairs. Acting on the response curves rather than on rendered pixels, the regularizer is independent of viewpoint, saturation, and scene content.

#### 4.2.3. Dual-Resolution Event-Stream Constraint

Event super-resolution networks operate in the image–time domain and do not explicitly enforce the multi-view geometry of the scene. The high-frequency details they add therefore need not be consistent across viewpoints. When $E^{H}$ serves as the only event supervision, the radiance field fits these mutually inconsistent details, and the reconstructed scene structures lose clarity across novel views.

We alleviate this inconsistency by supervising the synthesized events at the super-resolved resolution and the native resolution simultaneously. The first branch compares the synthesized events of Equation (9) against $E^{H}$ at the image resolution. The second branch constrains the rendering against the unmodified stream $E^{L}$, for which the rendered HDR images are first downsampled to the native resolution by a block-average pooling operator $A_{r}:R^{H\times W}\;\to \;R^{H_{L}\times W_{L}}$:

(13) $$\left(A_{r}\hat{I}\right)\left(i,j\right)=\frac{1}{r^{2}}\sum_{a=0}^{r-1}\sum_{b=0}^{r-1}\hat{I}\;\left(ri+a,\;rj+b\right),$$

where $r=H/H_{L}$ is the resolution ratio. The operator instantiates the downsampling operator D of Equation (6) and mirrors the spatial integration by which the event sensor produced $E^{L}$. For an event-pose $v\in V_{E}$ with temporal neighbor $v^{\prime }$, the LR events are synthesized from the downsampled renderings as in Equation (9):

(14) $${\hat{E}}_{v\to v^{\prime }}^{L}=\log\;\left(A_{r}{\hat{I}}_{v^{\prime }}^{\mathrm{HDR}}\right)-\log\;\left(A_{r}{\hat{I}}_{v}^{\mathrm{HDR}}\right),$$

where $V_{E}$ is the set of poses at which the event stream is supervised. High-frequency components introduced by the super-resolved stream are therefore constrained by the native-resolution measurement, encouraging the radiance field to retain only the details that are consistent across the two resolutions.

### 4.3. Optimization

In this section, we first assemble the supervision introduced above into the total training objective, and then describe the implementation details.

#### 4.3.1. Training Objective

The radiance field is trained under the joint supervision of the event stream, the LDR images, and the two corrective modules. At the pose pair $\left(v,v^{\prime }\right)$, the two branches of the dual-resolution constraint are combined into the event-stream loss:

(15) $$L_{e}\left(v,v^{\prime }\right)=\ell \;\left({\hat{E}}_{v\to v^{\prime }}^{H},\;E_{v\to v^{\prime }}^{H}\right)+\lambda_{\mathrm{LR}}\;\ell \;\left({\hat{E}}_{v\to v^{\prime }}^{L},\;E_{v\to v^{\prime }}^{L}\right),$$

where $\ell \left(\hat{x},x\right)={∥\hat{x}-x∥}_{1}+\lambda_{s}D_{\mathrm{SSIM}}\left(\hat{x},x\right)$, $\lambda_{\mathrm{LR}}$ balances the two branches, and the targets $E_{v\to v^{\prime }}^{H}$ and $E_{v\to v^{\prime }}^{L}$ are accumulated following Equation (10). The loss is averaged over the event-pose set, $L_{e}=\frac{1}{|V_{E}|}\sum_{v\in V_{E}}L_{e}\left(v,v^{\prime }\right)$. The image loss applies the same composite distance *ℓ* between the LDR prediction and the observation at the sampled image-pose *v*,

(16) $$L_{\mathrm{img}}=\ell \left({\hat{I}}_{v}^{\mathrm{LDR}},\;I_{v}^{\mathrm{LDR}}\right),$$

following [27]. Together with the color-correction terms of Equations (11)–(12), the total objective is

(17) $$L=L_{\mathrm{img}}+\lambda_{e}\;L_{e}+\lambda_{\mathrm{chr}}\;L_{\mathrm{chr}}+\lambda_{\mathrm{crf}}\;L_{\mathrm{crf}}+L_{\mathrm{reg}},$$

where $L_{\mathrm{reg}}$ collects the white-balance, rendering-uncertainty, and Gaussian-scale regularizers of [27], retained at their default weights. The Gaussian primitives G and the response curves $f_{c}$ are optimized jointly.

#### 4.3.2. Implementation Details

We implement the method on top of the official GaussHDR codebase [27] and run all experiments on a single NVIDIA RTX 4090 GPU. The super-resolution network S is instantiated with the public pre-trained BMCNet-ESR [18] checkpoint at scale factor r=4 without fine-tuning. Following the event-pose sampling protocol of [10,27], the event-pose set contains $|V_{E}|\;=\;35$ poses per scene. We set $C_{\mathrm{thr}}=0.1$, $\lambda_{s}=0.2$, $\lambda_{e}=0.1$, $\lambda_{\mathrm{LR}}=1$, and $\lambda_{\mathrm{chr}}=\lambda_{\mathrm{crf}}=1$. The saturation threshold of Equation (11) is 0.94, and Equation (12) uses M=1000 samples over the log-radiance range [−10,10]. Each training iteration samples one image-pose and one event-pose, at which the corresponding loss terms are evaluated. Optimization runs for 30,000 iterations with Adam, and all remaining settings follow the default schedule of [27].

## 5. Experiments

This section describes the experimental setup, compares our method against state-of-the-art baselines, and reports ablation studies.

### 5.1. Experimental Settings

We describe the benchmark, the evaluation metrics, and the baselines used for the quantitative comparisons and the ablation studies.

#### 5.1.1. Datasets

We conduct experiments on the EvHDR-NeRF synthetic benchmark [10], which provides time-synchronized event streams paired with multi-exposure RGB renderings for six indoor scenes. We follow its data-preparation protocol without modification. Each scene provides 35 viewpoints; for each scene, a separate radiance field is optimized on the 17 training views of the EvHDR-NeRF split, and all reported metrics are computed on the remaining 18 held-out test views of the same scene. The same split is used for all baselines. To emulate the resolution mismatch between the practical event and image sensors, the event stream is downsampled to $H_{L}\times W_{L}=200\times 200$ while the RGB renderings are retained at H×W=800×800.

#### 5.1.2. Evaluation Metrics

To assess both the fidelity and the perceptual quality of the reconstruction, we report PSNR [60], SSIM [60], and LPIPS [61]. Following standard practice [6,62,63], the HDR results are tone-mapped by the μ-law function before the metrics are computed:

(18) $$M\left(E\right)=\frac{\log(1+\mu E)}{\log(1+\mu )},$$

where μ=5000 is the tone-mapping parameter and *E* is the normalized HDR luminance in [0,1]. To assess the color cast directly (Section 5.3), we additionally report two color-specific measures on the tone-mapped results: the mean CIEDE2000 color difference $\Delta E_{00}$ [64], and the chromatic channel imbalance $\max_{c}b_{c}-\min_{c}b_{c}$, defined as the spread of the per-channel mean intensity bias $b_{c}=\bar{{\hat{I}}_{c}-I_{c}}$ over the RGB channels c∈{R,G,B}, which measures a systematic color shift while remaining insensitive to a channel-neutral brightness offset.

#### 5.1.3. Baselines

We compare against three classes of baselines, categorized according to how they handle the resolution mismatch before or during 3D reconstruction.

- Baseline-A. The LDR images are downsampled to $H_{L}\;\times \;W_{L}$, an event-aided HDR radiance-field method is trained at the low resolution, and the rendered HDR field is bicubic-upsampled to H×W for evaluation, following the protocol of image-domain NeRF super-resolution [65]. We instantiate the method with EvHDR-GS [11], a representative event-aided HDR reconstruction method built on 3D Gaussian Splatting.
- Baseline-B. The LR event stream is super-resolved to H×W, and EvHDR-GS is then trained at the image resolution with the upsampled events. We instantiate the super-resolution network with EventZoom [16], a widely used event up-sampling network, and BMCNet [18], the current state of the art in event super-resolution.
- Baseline-C. The two streams are fused in 2D image space first, using EvTexture [58], an event-driven 2D video super-resolution network, to produce HR LDR images from the LR events and LR images; HDR-GS [9] is then trained on these images alone, without any event signal entering the 3D stage.

### 5.2. Comparison with Baselines

Table 1 reports the per-scene HDR and LDR metrics of all methods on the six benchmark scenes, together with the six-scene mean.

Our full method achieves the best mean value on all three HDR metrics and improves the mean HDR PSNR by 1.44 dB over the strongest baseline—Baseline-B instantiated with BMCNet—while keeping the LDR metrics at a comparable level. Baseline-A reconstructs the scene at the event resolution, and the bicubic upsampling of its renderings cannot recover the high-frequency detail lost in the downsampled images. Baseline-B with BMCNet benefits from the super-resolved events and is the strongest competitor, yet it inherits the color cast and the multi-view inconsistency analyzed in Section 4.2.2 and Section 4.2.3, which limit its HDR accuracy. Baseline-B with EventZoom and Baseline-C fall behind on both HDR and LDR metrics, indicating that the events upsampled by EventZoom and the images fused by EvTexture are not consistent across viewpoints and therefore mislead the optimization of the radiance field. The improvement over the strongest baseline is statistically significant: a two-sided Wilcoxon signed-rank test over the paired per-view HDR metrics of all six scenes rejects the equality of our method and Baseline-B with BMCNet for all three tone-mapped HDR metrics (p<0.001 for PSNR, SSIM, and LPIPS). Repeating the full method with three random seeds on *bear* yields an HDR PSNR standard deviation of 0.10 dB, an order of magnitude below the margins in Table 1, confirming that the per-scene single-run protocol is stable.

**Figure 3:**
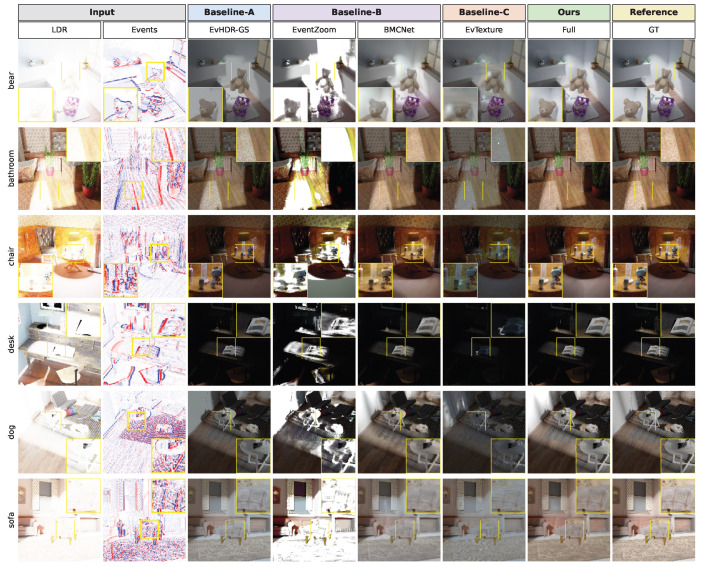
Qualitative comparison of tone-mapped HDR reconstructions on the six benchmark scenes. From left to right are the input LDR image and low-resolution event stream, the reconstructions of Baseline-A (EvHDR-GS [11]), Baseline-B (EventZoom [16] and BMCNet [18]), Baseline-C (EvTexture [58]) and our method, and the tone-mapped reference. Each row shows the same frame for all methods.

Figure 3 shows the tone-mapped HDR reconstructions of all methods on the six scenes. The qualitative results follow the same trend as the quantitative comparison. The reconstructions of Baseline-B with BMCNet exhibit a global color cast (*bear*, *desk*, *sofa*); EventZoom and EvTexture lead to over-darkened reconstructions with distorted structures; and the renderings of Baseline-A are blurry in regions with fine detail. In contrast, our method better preserves the reference color and high-frequency structure. The contribution of each module to this gap is quantified in Section 5.3.

### 5.3. Validation of Key Contributions

This section provides direct experimental evidence for the two modules introduced in Section 4.2. We show that the color correction module removes the global color cast introduced by the super-resolved events, and that the dual-resolution event-stream constraint alleviates the multi-view inconsistency of their high-frequency content.

#### 5.3.1. Effectiveness of Color Correction Module

We first validate the color correction module of Section 4.2.2. The variant (w/o color correction) disables the chrominance constraint of Equation (11) and the response-curve regularizer of Equation (12), while all other settings are kept identical to the full method. Table 2 reports the HDR metrics averaged over all six scenes.

**Figure 4:**
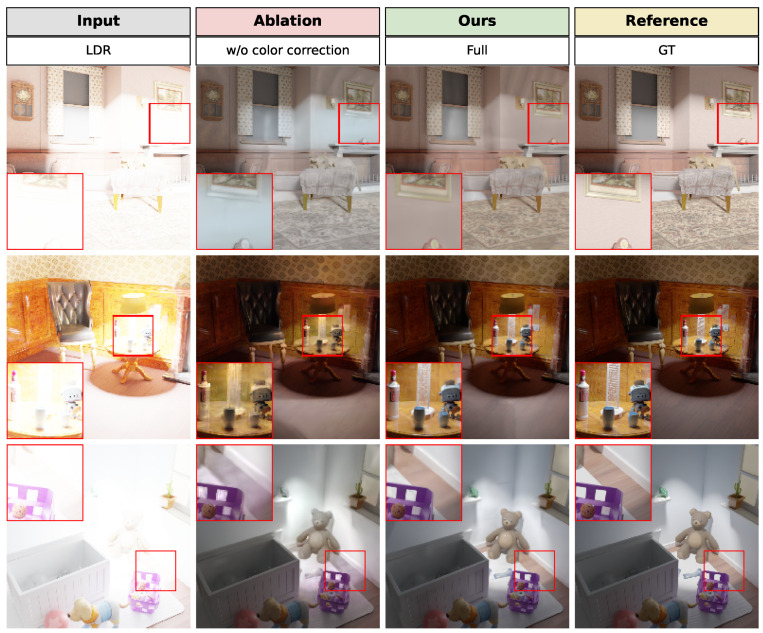
Qualitative effect of the color correction module. The *w/o color correction* variant exhibits a clear color cast in saturated and near-saturated regions, while the full method restores the chrominance of the reference. The rows show the *sofa*, *chair*, and *bear* scenes.

The color correction module anchors the rendered radiance to the chrominance cues of the LDR images, reducing the global color cast introduced by the super-resolved events. It improves the six-scene mean HDR PSNR by 2.11 dB and also improves the mean HDR SSIM and LPIPS. Figure 4 shows the qualitative effect. Without the color correction module, the reconstructions exhibit a clear color cast in saturated and near-saturated regions, while the full module restores the chrominance of the reference.

To quantify the color cast directly, Table 3 reports two color-specific measures computed between the tone-mapped HDR reconstruction and the tone-mapped reference: the mean CIEDE2000 color difference $\Delta E_{00}$, and the chromatic channel imbalance $\max_{c}b_{c}-\min_{c}b_{c}$, i.e., the spread of the per-channel mean intensity bias $b_{c}=\bar{{\hat{I}}_{c}-I_{c}}$ over c∈{R,G,B} (on the [0,1] scale). An unequal channel bias provides a simple measure of systematic global color imbalance, whereas a channel-neutral bias mainly reflects an overall luminance offset. The color correction module lowers the six-scene mean $\Delta E_{00}$ from 3.38 to 2.18 and the mean chromatic imbalance from $1.93\times 10^{-2}$ to $0.07\times 10^{-2}$, which directly quantifies the removal of the global color cast.

#### 5.3.2. Effectiveness of Dual-Resolution Event-Stream Constraint

We then validate the dual-resolution event-stream constraint of Section 4.2.3. The variant (w/o dual-resolution) supervises the synthesized events only at the super-resolved resolution, corresponding to $\lambda_{\mathrm{LR}}=0$ in Equation (15), while all other settings, including the full color correction module, are kept identical. Table 2 reports the HDR metrics.

**Figure 5:**
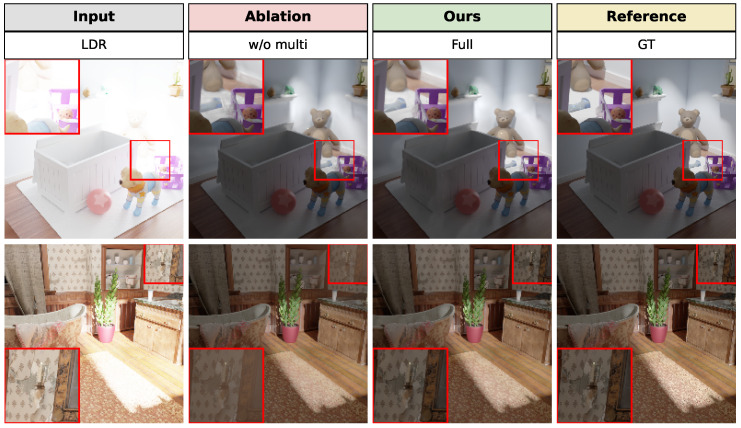
Qualitative effect of the dual-resolution event-stream constraint. The *w/o dual-resolution* variant loses clarity and exhibits chromatic drift inside the saturated regions, while the full method recovers structures that match the reference. The rows show the *bear* and *bathroom* scenes.

The native-resolution branch constrains the super-resolved supervision with the original event measurement, penalizing the high-frequency components that are not supported at the native resolution. This branch improves the six-scene mean HDR PSNR, SSIM, and LPIPS. However, because the PSNR gain is not uniform across individual scenes, we attribute its primary benefit to improved structural and perceptual consistency. Figure 5 shows the qualitative effect. The variant (w/o dual-resolution) reconstructions lose clarity and exhibit chromatic drift inside the saturated regions, while the full configuration recovers structures that better match the reference.

### 5.4. Ablation Study

This section studies the effect of the event contrast threshold $C_{\mathrm{thr}}$ and the robustness of our method to the event–image resolution gap.

#### 5.4.1. Event Contrast Threshold

The contrast threshold $C_{\mathrm{thr}}$ sets the scale at which the event signal is discretized in Equation (10). A threshold that is too small lets sensor noise enter the loss as if it were real signal, while a threshold that is too large suppresses the events triggered by real contrast changes. Table 4 sweeps $C_{\mathrm{thr}}$ over the range observed in practice on the *bear* scene, holding all other settings at their default values.

The reconstruction quality varies only mildly over the sweep, with all three HDR metrics degrading slightly as the threshold grows. The method is therefore not sensitive to the choice of $C_{\mathrm{thr}}$ within the practical range, and we keep the default threshold in all other experiments.

#### 5.4.2. Robustness to the Event–Image Resolution Gap

The benchmark in Section 5.2 fixes the event–image resolution gap at ×4. We further evaluate our method under a milder and a more aggressive gap, varying the resolution ratio r∈{2,4,8}. The image stream is kept at 800×800, and the event resolution is set to 400×400, 200×200, and 100×100, respectively. Each configuration applies the super-resolution network at the matching scale factor and is retrained under the same schedule. Table 5 reports the HDR metrics on the *bear* scene.

**Figure 6:**
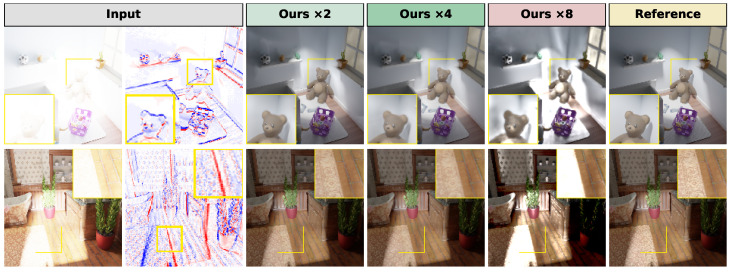
Qualitative results of our method at different resolution gaps on the *bear* scene. From left to right are the input (LDR image and native low-resolution event stream), our reconstructions at resolution ratios ×2, ×4, and ×8, and the tone-mapped reference. The yellow boxes mark a saturated patch, and the bottom insets show a 2× zoom of the same patch.

The reconstruction quality is stable between ×2 and ×4 and degrades sharply at ×8. The degradation stems from three interacting factors: aggressive downsampling removes a large fraction of the high-frequency event information and leaves the inverse problem underdetermined; the pretrained event super-resolution prior must hallucinate more of the missing detail and produces high-frequency events that are unreliable and inconsistent across viewpoints; and the native-resolution branch, which only constrains the block-averaged event signal, can reject some of this inconsistent content but cannot recover spatial details absent from the native measurements. The radiance field therefore receives conflicting high-resolution supervision together with insufficient native-resolution evidence, producing structural ambiguity, color deviation, and loss of fine detail. Figure 6 visualizes this trend. At ×2 and ×4 the reconstruction closely follows the tone-mapped reference, while at ×8 the super-resolved events carry high-frequency noise that the native-resolution branch can no longer absorb, and the reconstruction loses both color accuracy and fine detail.

#### 5.4.3. Loss Weights

The three loss weights introduced by our method, $\lambda_{\mathrm{LR}}$, $\lambda_{\mathrm{chr}}$, and $\lambda_{\mathrm{crf}}$, are all fixed to 1 without per-scene tuning. Table 6 varies each weight over {0.1,0.5,2} on the bear scene while holding the other two at the default. Across the whole sweep the HDR PSNR stays within 1.05 dB of the default configuration, and HDR SSIM and LPIPS vary only marginally, so the reconstruction quality plateaus around the default and the method does not require loss-weight tuning.

## 6. Limitations and Future Work

In this section, we first discuss the limitations of the proposed framework and then outline the directions in which it can be extended.

### 6.1. Limitations

The proposed framework has three main limitations. First, the event super-resolution network S is frozen during reconstruction and is not adapted to the target scene. Second, as shown in Section 5.4.2, performance degrades under very large resolution gaps, such as the ×8 setting, where the super-resolved events become too unreliable to provide stable 3D supervision. Third, the color correction module assumes a color event sensor with an RGBG Bayer pattern and is therefore not directly applicable to monochrome event cameras, whose adaptation would require a different event–image formation model and a modified color correction mechanism.

### 6.2. Future Work

Future work may jointly optimize the event super-resolution network and the radiance field, with the color correction module and the dual-resolution constraint in the loop, allowing the 2D prior to adapt to the 3D reconstruction objective. Another direction is progressive multi-scale reconstruction, which would extend the dual-resolution constraint to a genuinely multi-resolution one and could bridge large resolution gaps through intermediate event resolutions.

## 7. Conclusions

We presented a framework for HDR radiance-field reconstruction from resolution-mismatched event–image inputs, in which high-resolution single-exposure LDR images are paired with a low-resolution event stream from a hybrid camera rig. By combining a pretrained 2D event super-resolution prior with a color correction module and a dual-resolution event-stream constraint, the framework addresses the global color cast and the view-inconsistent high-frequency artifacts that arise when 2D event super-resolution directly supervises 3D HDR reconstruction. On the EvHDR-NeRF benchmark, the proposed method achieves higher six-scene mean HDR fidelity than the strongest baseline and improves the mean structural and perceptual metrics. These results indicate that transferring a 2D event super-resolution prior into 3D reconstruction requires correcting both the color and the multi-view consistency of the super-resolved events rather than treating event super-resolution as a self-contained preprocessing step.

## Figures and Tables

**Table 1:** Quantitative comparison with the baselines on the EvHDR-NeRF benchmark (30,000 iterations). Per-scene HDR and LDR metrics are reported together with the six-scene mean. Best results are in **bold**, second-best results are underlined. The value after ± on each **Mean** row is the standard deviation across the six scenes. ↑ and ↓ indicate that higher and lower values are better, respectively.

**Category**	**Method**	**Scene**	HDR (Tone-Mapped)			LDR		
			PSNR ↑	SSIM ↑	LPIPS ↓	PSNR ↑	SSIM ↑	LPIPS ↓
Baseline-A (Bicubic)	EvHDR-GS [11]	bear	21.38	0.9603	0.0445	42.53	0.9894	0.0050
		bathroom	29.33	0.9536	0.0265	36.78	0.9651	**0.0113**
		chair	24.28	0.9098	0.0909	33.24	0.9468	0.0257
		desk	34.05	0.9595	0.0369	38.37	0.9688	**0.0139**
		dog	23.80	0.9440	0.0620	38.62	**0.9876**	**0.0044**
		sofa	19.81	**0.9187**	**0.0708**	42.50	**0.9856**	**0.0062**
		**Mean**	25.44± 5.32	0.9410± 0.0217	0.0553± 0.0238	38.67± 3.54	0.9739± 0.0167	0.0111± 0.0081
Baseline-B (Event-SR)	EventZoom [16]	bear	14.14	0.8407	0.1984	41.72	0.9877	0.0067
		bathroom	16.58	0.6614	0.2085	35.74	0.9597	0.0151
		chair	15.03	0.7541	0.2569	33.84	0.9482	0.0236
		desk	14.25	0.7850	0.2562	37.80	0.9650	0.0168
		dog	13.92	0.8357	0.1919	38.34	0.9865	0.0052
		sofa	15.52	0.7396	0.2186	41.51	0.9796	0.0121
		**Mean**	14.91± 1.02	0.7694± 0.0671	0.2217± 0.0284	38.16± 3.12	0.9711± 0.0160	0.0133± 0.0068
	BMCNet [18]	bear	27.79	0.9647	0.0590	42.58	0.9894	**0.0048**
		bathroom	31.97	0.9505	0.0285	36.80	**0.9652**	0.0115
		chair	29.13	0.9250	0.1019	33.68	0.9488	0.0226
		desk	34.19	0.9653	0.0531	38.63	**0.9691**	0.0140
		dog	**25.81**	0.9286	0.0876	**38.69**	**0.9876**	0.0045
		sofa	**24.97**	0.9033	0.1042	42.85	0.9854	0.0067
		**Mean**	28.98± 3.57	0.9396± 0.0247	0.0724± 0.0303	38.87± 3.49	0.9743± 0.0161	0.0107± 0.0070
Baseline-C (2D-Fusion)	EvTexture [58]	bear	20.42	0.8821	0.1544	36.25	0.9430	0.0206
		bathroom	22.39	0.7645	0.2168	31.14	0.8931	0.0478
		chair	20.09	0.8160	0.2204	31.65	0.8957	0.0511
		desk	23.76	0.8775	0.1520	33.08	0.9168	0.0348
		dog	17.52	0.7980	0.2343	30.29	0.9256	0.0332
		sofa	16.40	0.7400	0.2305	35.89	0.9342	0.0296
		**Mean**	20.10± 2.80	0.8130± 0.0581	0.2014± 0.0379	33.05± 2.51	0.9180± 0.0203	0.0362± 0.0114
**Ours**	**full**	bear	**29.83**	**0.9749**	**0.0393**	**42.88**	**0.9895**	0.0055
		bathroom	**35.02**	**0.9620**	**0.0221**	**37.03**	0.9650	0.0123
		chair	**32.81**	**0.9621**	**0.0497**	**35.31**	**0.9535**	**0.0191**
		desk	**39.03**	**0.9805**	**0.0254**	**38.75**	**0.9691**	0.0147
		dog	25.12	**0.9533**	**0.0527**	38.44	0.9873	0.0049
		sofa	20.72	0.9087	0.0821	**42.95**	**0.9856**	0.0067
		**Mean**	**30.42**± 6.69	**0.9569**± 0.0256	**0.0452**± 0.0219	**39.23**± 3.11	**0.9750**± 0.0146	**0.0105**± 0.0058

**Table 2:** Validation of the color correction module and the dual-resolution event-stream constraint (30,000 iterations), reported as the six-scene mean. Each variant disables one module while keeping all other settings identical to the full method. Best results are in **bold**, second-best results are underlined. ↑ and ↓ indicate that higher and lower values are better, respectively.

Variant	HDR PSNR↑	HDR SSIM↑	HDR LPIPS↓
w/o color correction	28.31	0.9315	0.0781
w/o dual-resolution	29.58	0.9523	0.0484
**Ours (full)**	**30.42**	**0.9569**	**0.0452**

**Table 3:** Direct color-cast evaluation of the color correction module (30,000 iterations) on all six scenes. $\Delta E_{00}$ is the mean CIEDE2000 color difference; the chromatic channel imbalance ($\times 10^{-2}$) is the spread of the per-channel intensity bias. Lower is better for both; best per column in **bold**. ↑ and ↓ indicate that higher and lower values are better, respectively.

Metric	Variant	Bear	Chair	Desk	Dog	Sofa	Bathroom	Mean
$\Delta E_{00}↓$	w/o color correction	4.11	3.06	0.97	3.57	6.01	2.54	3.38
	**Ours (full)**	**2.05**	**1.60**	**0.41**	**2.39**	**5.47**	**1.19**	**2.18**
imbal.	w/o color correction	2.06	0.82	0.38	0.34	5.90	2.09	1.93
$(\times 10^{-2})↓$	**Ours (full)**	**0.01**	**0.08**	**0.02**	**0.05**	**0.15**	**0.11**	**0.07**

**Table 4:** Effect of the event contrast threshold $C_{\mathrm{thr}}$ on reconstruction quality. ↑ and ↓ indicate that higher and lower values are better, respectively.

$C_{\mathrm{thr}}$	0.05	0.10	0.20	0.30
HDR PSNR↑	29.95	29.83	29.54	29.13
HDR SSIM↑	0.9816	0.9749	0.9510	0.9379
HDR LPIPS↓	0.0310	0.0393	0.0401	0.0414

**Table 5:** Robustness to the event–image resolution gap. HDR (tone-mapped) results of our full method at resolution ratios r∈{2,4,8}. ↑ and ↓ indicate that higher and lower values are better, respectively.

Ratio *r*	PSNR ↑	SSIM ↑	LPIPS ↓
×2	29.77	0.9766	0.0340
×4 (default)	29.83	0.9749	0.0393
×8	15.41	0.8641	0.1787

**Table 6:** Loss-weight sweep on the bear scene. ↑ and ↓ indicate that higher and lower values are better, respectively

Weight	Value	HDR PSNR↑	HDR SSIM↑	HDR LPIPS↓
$\lambda_{\mathrm{LR}}$	0.1	30.53	0.9729	0.0447
	0.5	29.85	0.9742	0.0403
	2.0	28.78	0.9741	0.0371
$\lambda_{\mathrm{chr}}$	0.1	29.62	0.9747	0.0385
	0.5	29.85	0.9747	0.0396
	2.0	29.71	0.9741	0.0395
$\lambda_{\mathrm{crf}}$	0.1	29.64	0.9750	0.0382
	0.5	29.63	0.9746	0.0398
	2.0	29.73	0.9749	0.0363
Default	all =1	29.83	0.9749	0.0393

## Data Availability

The dataset presented in this study is openly available in Ref. [10].
